# Schistosomiasis Coinfection in Children Influences Acquired Immune Response against *Plasmodium falciparum* Malaria Antigens

**DOI:** 10.1371/journal.pone.0012764

**Published:** 2010-09-15

**Authors:** Tamsir O. Diallo, Franck Remoue, Lobna Gaayeb, Anne-Marie Schacht, Nicole Charrier, Dick De Clerck, Jean-Pierre Dompnier, Sophie Pillet, Olivier Garraud, Abdoulaye A. N'Diaye, Gilles Riveau

**Affiliations:** 1 Inserm Unité 547, Institut Pasteur de Lille, Lille, France; 2 ONG ESPOIR Pour La Santé, Laboratoire de Recherches Médicales, B.P. 226, Saint-Louis, Sénégal; 3 Institut de Recherche pour le Développement (IRD) - UR 016 “Caractérisation et Contrôle des Populations de Vecteurs” - BP 64501 - 34394, Montpellier, France; 4 GIMAP, EA3064, Université Jean Monnet, Saint Etienne, France; Karolinska Institutet, Sweden

## Abstract

**Background:**

Malaria and schistosomiasis coinfection frequently occurs in tropical countries. This study evaluates the influence of *Schistosoma haematobium* infection on specific antibody responses and cytokine production to recombinant merozoite surface protein-1-19 (MSP1-_19_) and schizont extract of *Plasmodium falciparum* in malaria-infected children.

**Methodology:**

Specific IgG1 to MSP1-_19_, as well as IgG1 and IgG3 to schizont extract were significantly increased in coinfected children compared to *P. falciparum* mono-infected children. Stimulation with MSP1-_19_ lead to a specific production of both interleukin-10 (IL-10) and interferon-γ (IFN-γ), whereas the stimulation with schizont extract produced an IL-10 response only in the coinfected group.

**Conclusions:**

Our study suggests that schistosomiasis coinfection favours anti-malarial protective antibody responses, which could be associated with the regulation of IL-10 and IFN-γ production and seems to be antigen-dependent. This study demonstrates the importance of infectious status of the population in the evaluation of acquired immunity against malaria and highlights the consequences of a multiple infection environment during clinical trials of anti-malaria vaccine candidates.

## Introduction

*Plasmodium falciparum* (*Pf*) malaria remains one of the major public health issues in tropical countries and the vast majority of childhood deaths due to malaria occur in sub-Saharan Africa [1].

Protective immunity to *Pf* malaria is slowly acquired after several infections and is dependent on the intensity and duration of the individual exposure to the parasite [2]. The *P. falciparum* merozoite surface protein 1 (MSP1) and especially its highly conserved C-terminal EGF-like module pair, known as MSP1-_19_ is one of leading candidate antigens for a vaccine against the malaria parasite blood stage [3], [4]. In infected humans, humoral immune responses to blood stage parasites play a primary role in providing protection against malaria [5] and they are largely dependent on cytophilic type immunoglobulin (Ig) antibodies (Abs) such as IgG1 and IgG3 isotypes [4]. In addition, a specific cellular immune response and its associated cytokines, play a key protective or pathological role during malaria. Some cytokines, such as interferon-γ (IFN-γ) and Interleukin-10 (IL-10), are known to be directly involved in the production of specific isotypes of anti-*P. falciparum* Ab responses [6]. Hartgers *et al*. recently demonstrated that in Ghanaian school children, there was an increase in specific IL-10 production in helminth-infected individuals, compared to non-helminth infected [7]. Therefore, the regulation of the specific production of cytokines induced by such parasite infection could have a substantial impact on the development of malaria protection, as well as on its pathological consequences [7]–[8].

Intrinsic features of the immune system that develop with age but also with the presence of other chronic infections may influence malaria immunity [9]–[11]. Indeed, a chronic coinfection, such as schistosomiasis, may have a key impact on the acquired immune response to *Plasmodium* infection [9]. Previous studies, relating the complexity of interactions between host response to helminths and malaria infection, suggested possible consequences on age-dependent malaria morbidity [12]–[14]. In addition, the presence of *Schistosoma* coinfection during uncomplicated *P. falciparum* malaria unbalances the regulation of the associated inflammatory response [15]. Coinfection with helminthic parasites could then constitute a confusing factor in the assessment of efficacy of malaria-control intervention, including vaccine clinical trials [7], [12].

The present study evaluates the impact of coinfection by *S. haematobium (Sh)* on the specific isotype Ab response and its associated cytokine production to PfMSP1-_19_ and to schizont extracts of *P. falciparum* asexual blood stage antigens (Ags) in children living in a particular malaria endemic area, where schistosomiasis appeared 15 years ago [15].

## Materials and Methods

### Subjects

The studied population was a cohort of 79 malaria infected children living in the same area of the Senegal River basin (villages of Lampsar, Taba Tache and Taba Dar Salam), as previously described [16]. Two groups were identified in this cohort: children infected by *P. falciparum* without a confirmed schistosomiasis (*Pf*) (n = 40; age mean = 11 years; range: 7–15; villages of Taba Tache and Taba Dar Salam) and malaria infected children presenting a coinfection with *S. haematobium* (*Pf-Sh*) (n = 39; age mean = 10 years; range: 7–15; villages of Lampsar). Comparable mean age and sex ratio were obtained. Absence of clinical morbidity for malaria and schistosomiasis was a criterion of selection. The detection of schistosomiasis infection was performed using the referent parasite criteria (presence of eggs in urine or/and in feces). Schistosoma infected patients presenting pathological cases of schistosomiasis were treated but not included in the study. The Taba Tache and Taba Dar Salam villages are located down-river from Diama dam on the Senegal River where schistosomiasis is absent as previously described (13–14) and as confirmed by the present study.

### Diagnosis

#### *Pf* infection

*Pf* infection was detected by Quantitative Buffy Coat (QBC) (Becton Dickinson) and parasites were then counted and identified on blood smears. These tests are commonly used in malaria endemic areas and blood smears represent the referent criterion to detect malaria infection [17]. Only red blood cells infected by *Pf* were observed on positive slides. No other *Plasmodium* species infection was detected. The studied population was considered positive for malaria when *Pf* was detected with the QBC test and confirmed by blood smear observation, over a one-month period. In coinfected children, the mean parasitaemia of *P. falciparum* was not significantly different from the respective *Pf* mono-infected groups (Mann–Whitney *U*-test). Mean of parasitaemia was 1617 per mm^3^/blood for *Pf* infected children versus 340 per mm3/blood for coinfected children. During pre-selection, subjects showing high positivity in QBC (+ + +) were excluded and immediately treated for malaria. Children presenting clinical symptoms of mild or severe malaria morbidity were not selected. The studied population did not therefore present clinical symptoms of malaria morbidity. None of the selected children had received anti-malaria treatment in the month preceding the study.

#### *Schistosoma* heamatobium infection

The presence of *Sh* eggs was evaluated in three samples of urine (urine filtration) using microscopy. In the studied area and population, no *Schistosoma mansoni (Sm)* infection was detected using Kato–Katz method.

In the villages of Taba Tache and Taba Dar Salam, schistosomiasis infection has never been previously detected (16; D. De Clercq, unpublished data). In addition to the complete absence of schistosome eggs in faeces and urine, we have confirmed the absence of *Schistosoma* infection in these villages by evaluating the schistosome worm circulating anodic antigen (CAA) in sera, as previously described [16].

In Lampsar village, the presence of *Sm* and *Sh* eggs was evaluated in three samples of faeces (Kato–Katz method) or urine (urine filtration), respectively. The intensity mean of urinary *Sh* was 57 eggs/10 ml of urine.

None of the selected children had received anti-schistosome treatment in the previous 6 months.

The study followed ethical principles and was approved by the ethics committee of the Senegalese Ministry of Health. Written informed consent was obtained from the study population by their parents or guardians.

### *P. falciparum* and schistosome antigens

The *P. falciparum* antigens were recombinant PfMSP1-_19_ and schizont total antigens. PfMSP1-_19_ was produced in *Pichia pastoris* as a hexa-His fusion [18]. PfMSP1-_19_ was provided by J. McCormick and W. Morgan from National Institute for Medical Research (London, UK). The schizont antigenic preparation of *P. falciparum* is a soluble extract of schizont lysate obtained from infected erythrocyte cultures [19].

Soluble Egg Antigen (SEA) is a total soluble extract of schistosome eggs obtained from experimental infection in guinea pigs by the Antigen Production Laboratory at the Pasteur Institute of Lille, France.

### Human Ab levels

Specific antibody levels to PfMSP1-_19_, schizont extract or SEA in sera were determined by enzyme-linked immunosorbent assay (ELISA).

PfMSP1-_19_ protein (0.5 µg/ml), schizont extract (0.5 µg/ml) or SEA (5 µg/ml) were coated on 96-well plates (Nunc, Denmark) for 2 h 30 at 37°C. Plates were then blocked in PBS containing 0.5% gelatin (Merck, Germany). The sera diluted in PBS/Tween were incubated at 4°C overnight at a 1/100 dilution for IgG2, IgG3 and IgG4, 1/50 for IgA and IgE, and at 1/1,000 for IgG1 detection to schizont extract. For MSP1-_19_ antigen, a 1/100 dilution for IgG2, IgG3, IgG4 and IgA, 1/50 for IgE and 1/1,500 for IgG1 were used and for SEA, a 1/1,000 dilution for IgG1 and 1/100 for IgG3. Corresponding biotinylated mAbs to human Ig isotypes (SBA, AL or BD Pharmingen, CA, USA), were then incubated at a 1/1,000 dilution for IgG2, IgG4; 1/2,000 for IgG1, 1/750 for IgG3; 1/1,500 for IgA and 1/250 for IgE (1 h 30 mn at 37°C). Streptavidin-horseradish peroxidase-conjugated for IgG1, IgG2, IgG3, IgG4 and IgA detection (1/1,500, 30 min at 37°C; Amersham, Les Ulis, France) and ExtrAvidinR peroxidase conjugate for IgE detection (1/5,000, 30 min at 37°C; Sigma, St-Louis, MO) were then added. These ELISA conditions have been determined according to the results of previous experiences. Colorimetric development was allowed by means of ABTS (2,2′-azino-bis (3-ethylbenzothiazoline 6-sulphonic acid) diammonium; Sigma) in 50 mM citrate buffer (pH = 4 containing 0.003% H_2_O_2_), and absorbance (OD) was measured at 405 nm for IgG1, IgG2, IgG3, IgG4 and IgA. IgE detection was revealed by OPD (1,2 - Phenylene Diamine Dihydrochloride, Dako; Glostrup, Denmark). OPD was added for 30 minutes, followed by the addition of 2N H_2_SO_4_ to stop the reaction and OD was measured at 492 nm. The same ELISA procedures were performed in parallel for 30 sera from uninfected European subjects used as negative controls. Individual results were expressed as ΔOD value calculated for each isotype test according to the formula: ΔOD  =  ODx-ODn, where ODx is the individual OD value of infected patient and ODn was the arithmetic mean of individual OD value for the 30 uninfected control individuals (ODn value for IgE = 0.107; IgG1 = 0.235; IgG2 = 0.123; IgG3 = 0.124; IgG4 = 0.111, IgA = 0.159 using schizont extract; IgE = 0.09, IgG1 = 0.156, IgG2 = 0.121, IgG3 = 0.128, IgG4 = 0.120, IgA = 0.181 using MSP1-_19_; IgG1 = 0.151, IgG3 = 0.147 using SEA).

### Adsorption procedure of sera with schistosome antigens

Individual sera of *Pf/Sh* coinfected subjects were diluted with PBS/Tween solution and incubated with schistosomes Soluble Egg Antigens (SEA) at a final concentration of 200 µg/ml. The mixture (sera-SEA) was incubated with gentle shaking for 5 h at room temperature to allow the binding of specific Abs to SEA. Specific IgG1 and IgG3 responses to SEA, to schizont extract of *Pf* or to PfMSP1-_19_ were evaluated by ELISA (as described above) before and after this adsorption procedure.

### Whole blood culture and cytokine production measurement

Sterile conditions were maintained during the whole blood culture procedure, as originally described [20]. After white blood cell count, blood samples were diluted in RPMI-1640 medium (Gibco, France) supplemented with 2 mM L-glutamate, 8 µg/ml Gentaline, and 1 mM Sodium Pyruvate in order to obtain 2×10^6^ mononuclear cells/ml. Blood cells were then distributed at the rate of 1 ml/well in 24-well plates (Nunc). Antigens were added to each culture well to a final concentration of 1 µg/ml for MSP1-_19_ and 10 µg/ml for schizont extract. After 3 days of incubation at 37°C in 5% CO2, the content of each culture well was centrifuged, and supernatants were harvested and conserved at −80°C. Supernatant concentrations of IL-10, IL-12, IL-13, IFN-γ and TGF-β were measured at day 3 by means of ELISA kits according to the manufacturer's instructions (Immunotech, France). Specific cytokine production after Ag stimulation were expressed as picograms, nanograms or International Units (IU) per millilitre after the subtraction of the amount detected in medium control cultures (non-stimulated conditions) for each individual. Identical culture conditions were assessed with mononuclear cells of 30 uninfected control individuals in which no cytokine production was detected after Ag stimulation (data not shown).

### Statistical analysis

All data were analysed with Graph Pad Prism (Graph Pad Software, CA, USA). After verifying that values did not assume a Gaussian distribution, the nonparametric Mann-Whitney *U-test* was used to compare values between both independent groups. Correlation between specific Ab levels and the intensity of *P. falciparum* infection was analysed using Spearman's rank correlation coefficient. All differences were considered significant at *p*<0.05.

## Results

### Specific Ab responses to PfMSP1-_19_ and schizont extract

**Figure 1:**
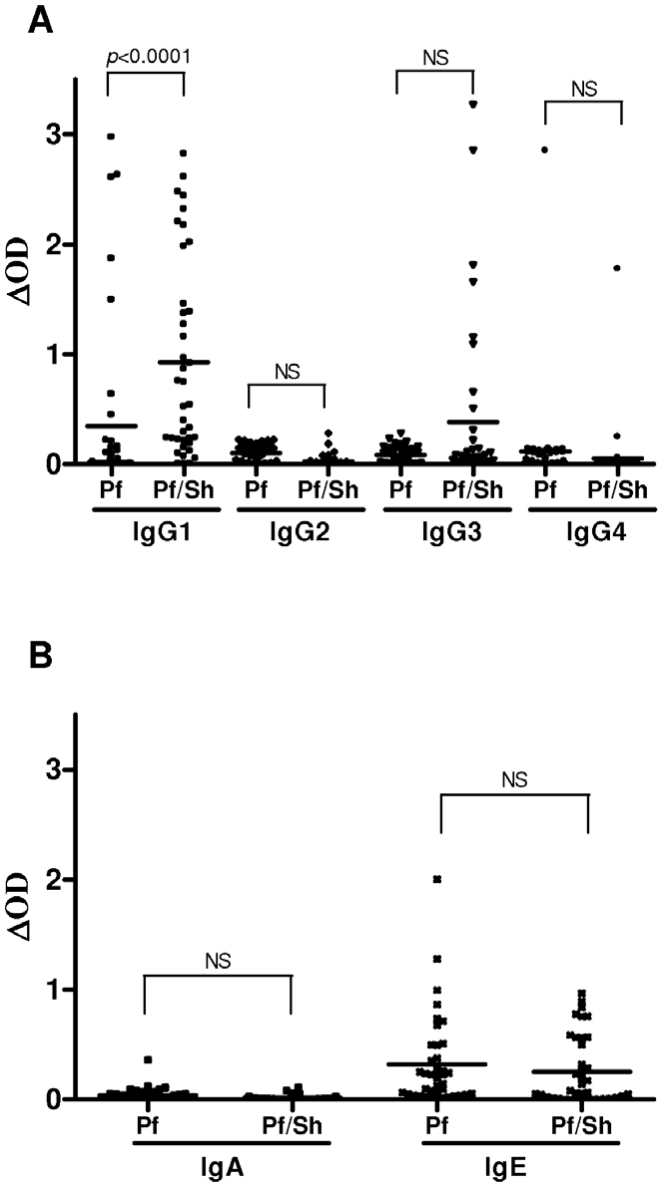
Specific Ab responses to PfMSP1-_19_ in children infected by *P. falciparum* (*Pf* - n = 40) and coinfected with *S. haematobium* (*Pf-Sh* - n = 39) as determined by ELISA. Results represent individual values of ΔOD and bars are the arithmetic mean for each group. Statistical significance between each group is indicated. (A) isotypes IgG1, IgG2, IgG3 and IgG4, (B) isotypes IgA and IgE.

The comparison of specific isotype Ab response to MSP1-_19_ between children having been solely infected by *P. falciparum* (*Pf)* and those coinfected with *S. haematobium* (*Pf/Sh*) was assessed (Figure 1). IgG1 Ab levels were significantly higher in *Pf/Sh*-infected children, compared to *Pf-*infected children (P<0.05) (Figure 1A). The specific IgG3 response was detected in a few *Pf/Sh*-infected children but no statistical difference was observed between both groups (Figure 1A). No relevant anti-MSP1-_19_ IgG2, IgG4 and IgA responses were observed in either group and the levels of specific IgE Ab were similar between *Pf*- and *Pf/Sh*-infected children (Figure 1A and 1B).

**Figure 2:**
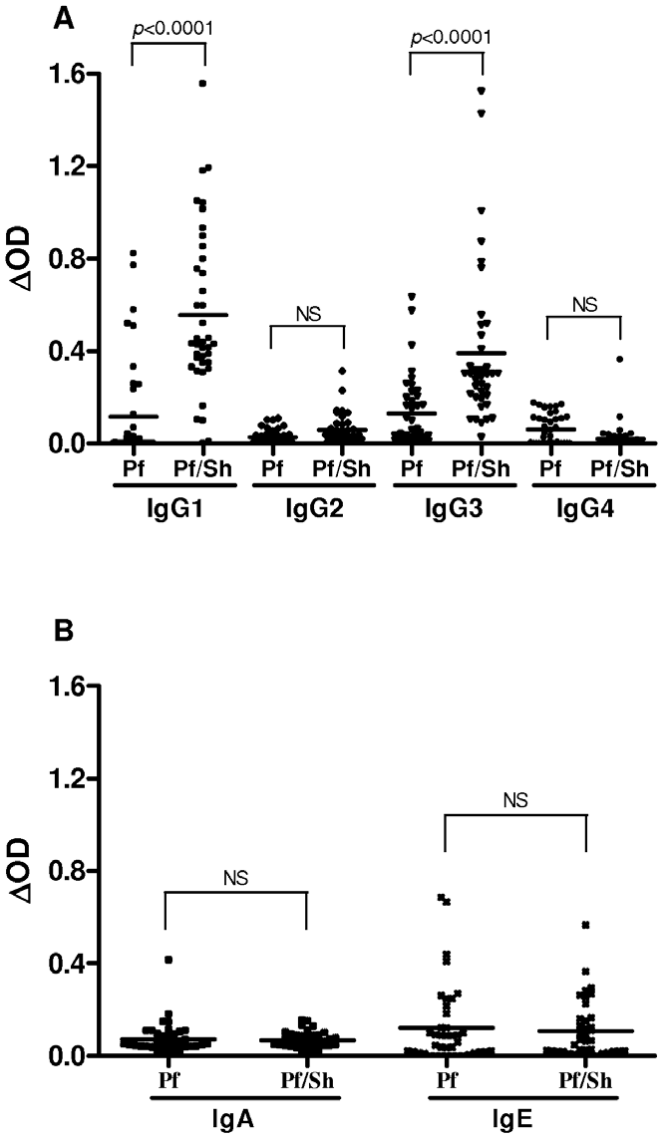
Specific Ab responses to schizont extract in children infected by *P. falciparum* (*Pf* - n = 40) and coinfected with *S. haematobium* (*Pf-Sh* - n = 39) as determined by ELISA. Results represent individual values of ΔOD and bars are the arithmetic mean for each group. Statistical significance between each group is indicated. (A) isotypes IgG1, IgG2, IgG3 and IgG4, (B) isotypes IgA and IgE.

To evaluate the antigenic restriction of the observed isotype variations according to infectious status, Ab responses against whole schizont extract were also assessed. As observed with the MSP1-_19_ Ag, specific IgG2, IgG4, IgA, and IgE responses to total schizont extract were very low or similar between *Pf* and *Pf/Sh* groups (Figure 2A and 2B). In contrast, specific IgG1 and IgG3 Ab levels were significantly higher in *Pf/Sh*-infected children compared to *Pf*-infected group (Figure 2A). These results suggest that the observed difference in IgG1 responses is not restricted to MSP1-_19_ antigen but is also found with *P. falciparum* total extract.

### Absence of cross-reactivity of anti-*P. falciparum* IgG1 and IgG3 from coinfected patients with schistosome antigens

We cannot exclude that antigenic cross-reactions can at least partly account for increased levels of anti-*P. falciparum* IgG1 and IgG3 observed in *Pf/Sh* coinfected group. Adsorption experiments with total schistosome antigens (SEA) of sera from coinfected group were therefore performed in order to verify the possible presence of IgG1 and IgG3 Abs cross-reacting with schistosome and *P. falciparum* antigens.

First, results show a complete adsorption of anti-SEA IgG1 and IgG3 Abs after incubation of the *Pf/Sh* patient sera with SEA, indicating the efficiency of the experimental procedure (Table 1). Using the same individual samples, we have evaluated the specific IgG1 and IgG3 Ab levels against both *P. falciparum* Ags (schizont extract and MSP1-_19_) before and after Abs adsorption. The comparison shows that the mean OD values were similar between SEA adsorbed and non adsorbed sera for both schizont extract and MSP1-_19_ Ags (Table 1). These data show that increased levels of IgG1 and IgG3 isotypes recognizing malaria antigens in the coinfected group could not be explained by a cross reactivity to Ags from both parasites.

**Table 1:** Absence of cross reactivity of anti-*P. falciparum* IgG1 and IgG3 from coinfected patients with Schistosome antigens.

	Schistosome Ag		*P. falciparum* Ags			
	SEA		SE		PfMSP1-_19_	
	NA	A	N A	A	NA	A
**IgG1** a	1.024±0.222	0.019±0.09*	0.336±0.063	0.323±0.061	1.885±0.319	1.762±0.311
**IgG3**	0.413±0.115	0.013±0.004*	0.259±0.065	0.342±0.079	0.683±0.257	0.704±0.250

a : Results are mean ± SEM of individual specific production (after subtracting the amount detected in unstimulated medium control cultures for each individual).

Ag: Antigen.

SEA: Schistosoma Egg Antigens.

SE: Schizont Extract.

A: Adsorbed Sera with SEA.

NA: Non Adsorbed Sera with SEA.

*: p<0.0009.

### Influence of intensity of *P. falciparum* infection on the variation of anti-*P. falciparum* IgG1 and IgG3 levels observed between groups of children

The correlation between specific Ab levels and the parasitaemia due to *P. falciparum* infection was first analysed using Spearman's rank correlation coefficient, without taking into account the coinfection status. This analysis did not reveal any significant correlation (data not shown). This suggests that the observed quantitative difference in the anti- *P. falciparum* Abs responses between both groups were not due to differences in the intensity of blood malaria parasites but could be related to the influence of *Schistosoma* coinfection.

In addition, we have compared individual IgG1 and IgG3 responses against schizont Ags from both groups (coinfected *vs*. mono-infected) matched on the level of *Pf* parasitaemia. We have obtained 25 matched-pairs with parasitaemia values varying from 3 to 3780 infected erythrocytes per mm^3^ of blood. The Wilcoxon *t*-test was used to compare the means of the matched-pairs. Regarding Ab responses against schizont Ags, the obtained value was *t* = 3.75 for IgG1 (*p*<0.01) and *t* = 3.23 for IgG3 (*p*<0.001), indicating that both groups were significantly different regarding their IgG1 and IgG3 levels when patients were matched on their parasitaemia.

Taken together these results indicate that the level of *P. falciparum* parasitaemia should not be considered as a confounding factor implicated in the observed variation of specific IgG1 and IgG3 responses according to infectious status.

### Production of cytokines in response to PfMSP1-_19_ and schizont extract

The production of specific IFN-γ, IL-10, IL-12, IL-13 and TGF-β cytokines after *in vitro* stimulation of mononuclear cells from studied individuals by schizont or MSP1-_19_, was evaluated in children (Table 2). These cytokines have been selected according to previous studies showing their role in the immune response regulation during malaria infection and for some of them, their role in the regulation of Ab isotype production [7]. The specific production of IL-12, IL-13 and TGF-β to both *P. falciparum* antigens was not significantly different between *Pf* and *Pf/Sh* groups. In contrast, specific IL-10 and IFN-γ production after MSP1-_19_ or schizont extract stimulation, showed differences between both groups. IL-10 production in presence of both Ags was significantly higher in *Pf/Sh*-infected children than in *Pf*-infected children (*p* = 0.025, *p* = 0.002, respectively). As for specific IFN-γ secretion, difference between both groups was only observed for the schizont extract and was significantly higher in *Pf/Sh*-group compared to *Pf*-group (*p* = 0.02). Nevertheless, high IFN-γ production to MSP1-_19_ was observed in few coinfected children (12/39) but the mean value of IFN-γ secretion in this group was not statistically higher than the one observed in *Pf*-group (*p* = 0.282). Background production of IFN-γ and IL-10 (medium alone) was similar between both groups (IFN-γ: 1.25 [mean] ±2.38 [SEM] UI/ml in *Pf* group, and 4.95±10.71 UI/ml in *Pf/Sh* group; NS) (IL-10: 248.1±219 ng/ml in *Pf* group, and 185.2±140.3 ng/ml in *Pf/Sh* group; NS). These last results indicate that the differences of cytokine production according to infection status were observed only after the stimulation by *Plasmodium* antigens.

**Table 2:** Specific cytokine production to PfMSP1-_19_ and Schizont extract in children infected by *P. falciparum* (*Pf*, n = 40) or coinfected by *P. falciparum* and *S. haematobium* (*Pf-Sh,* n = 39).

	PfMSP1-_19_			Schizont Extract		
	*Pf*	*Pf*/*Sh*	P value^b^	*Pf*	*Pf*/*Sh*	P value^b^
**IL-10** a **(ng/ml)**	14.73±5.21	22.42±4.89	**0.025**	36.64±10.99	72.99±10.79	**0.002**
**IL-12 (pg/ml)**	17.24±5.63	16.82±6.06	0.207	20.47±7.47	26.23±8.72	0.386
**IL-13 (pg/ml)**	2.34±1.33	3.07±1.09	0.219	4.22±1.69	4.10±2.08	0.495
**IFN-γ (pg/ml)**	0.26±0.11	4.22±2.05	0.282	1.72±0.48	11.13±5.34	**0.020**
**TGF-β (pg/ml)**	6.72±1.83	5.23±1.89	0.286	12.65±2.31	10.81±1.60	0.259

a : Results are mean ± SEM of individual specific production (after subtracting the amount detected in unstimulated medium control cultures for each individual).

b : P value between *Pf* and *Pf/Sh* groups (nonparametric Mann-Whitney *U test*). Differences are considered significant for P<0.05.

## Discussion

Following the study of the influence of schistosomiasis coinfection on the regulation of inflammatory factors in uncomplicated *P. falciparum* malaria [16], we have here evaluated the influence of coinfection in children on the acquired specific immune response to total *Plasmodium* Ags extract (schizont stage) and to the specific PfMSP1-_19_ vaccine candidate. The greatest interest of this study is that the selected children lived in the same low malaria area (confirmed by the similar *Pf* parasitemia between groups), without clinical manifestations, but living in villages from the same studied area, where schistosomiasis was monitored since several years (13–14). In particular, it indicated that *Pf* mono-infected children have never been infected by *Schistosoma*. We demonstrated that *S. haematobium* coinfection influenced the humoral immune response against malaria antigens by specifically increasing IgG1 and IgG3 Abs levels. This increase was not dependent on the age, on the intensity of malaria infection or on a potential antigenic cross-reactivity. Indeed, the IgG1 and IgG3 responses to schizont extract were higher in *Pf/Sh*-infected children compared to the *Pf*-infected group. However, only specific IgG1 Ab response to the MSP1-_19_ antigen was significantly increased in coinfected children suggesting that the influence of coinfection could be antigen-dependent. During human malaria, IgG1 and IgG3 are thought to play a key role in protection [21], [22]. It is believed that these cytophilic IgG subclasses can neutralize parasites directly, by inhibiting parasite invasion or growth in erythrocytes, or indirectly by a mechanism involving cooperation between parasite–opsonising antibodies and monocytes, through binding to the Tcc receptor IIA [23]–[25]. The subclass response to MSP1-19 was biased towards IgG1 with a minor component from the IgG3 subclass [26]. In addition, the presence of specific IgG1 instead of IgG3 was associated with clinical protection against malaria infection [27], [28].

In the present study, we could also rule out the possible Abs cross reactivity between total *Sh* and *Pf* antigens, by assessing SEA adsorption experiments. In addition, statistical analysis showed the absence of an effect of the intensity of *P. falciparum* parasitaemia on these specific Ab responses to malaria antigens. Altogether, the data suggest that chronic schistosome infection in malaria-infected children could affect the specific immune response to *P. falciparum*. In particular, *S. haematobium* coinfection appeared to influence the specific isotype Ab responses involved in the development of protective immunity to *P. falciparum* infection. Moreover, we and others have described that *P. falciparum* coinfection also influenced the anti-schistosome immune response in *S. haematobium*-infected children [29], [30]. In this case, anti-schistosome IgG3 level was increased in coinfected individuals in contrast to the other IgG subclasses. In regard to our present results, the co-occurrence of schistosomiasis and malaria in individuals could have a particular effect on the IgG3 isotype regulation. The specific IgG3 response to MSP1-_19_ has been observed in 1/3 of coinfected children (12/39) whereas this isotype response was not detected in children infected only by *Pf*. Several immuno-epidemiological studies indicate that the presence of anti-MSP1-_19_ IgG3 response could be dependent to the studied site. For example, a very low IgG3 antibody response to MSP1-_19_ antigen was observed in some malaria endemic areas [26], [28], [31]. This difference might be due to different genetic background of these populations and/or to a different intensity of transmission in the studied areas or to environmental factors. Indeed, it was speculated that the differential evolution of plasma IgG1 and IgG3 antibodies resulted from a different catabolism and/or use of these antibodies [32]. Thus, history of malaria exposure, seasonal transmission and the short life of specific IgG3 to MSP1-_19_ antigen cannot be excluded to explain the presence or the absence of this specific IgG3 response. Nevertheless, our results indicate that the presence of specific IgG3 to MSP1-_19_ among some children living in an endemic malaria area was observed when schistosomiasis coinfection has been established. It could be of great interest to confirm the observations of our study in other coinfection contexts. Our study indicates that it will be crucial to systematically consider the coinfection status of patients in studies concerning the anti-malaria immunity. It strengthens the necessity to screen patients for helminth infections and their immune influence before and during malaria vaccine trials, as previously suggested [9].

We have demonstrated that the influence of *S. haematobium* infection on specific immune response to *Plasmodium* Ags involves specific cytokine production. Helminth infections seemed to influence IL-12, IL-13 and TGF-β production under stimulation with both malaria Ags. In contrast, specific IFN-γ and IL-10 production to the schizont extract were significantly higher in the coinfected group, which showed also a predominant anti-schizont IgG1 and IgG3 Ab levels. In addition, specific IL-10 production to MSP1-_19_ was significantly higher in the coinfected group compared to children only infected by *Pf*, as observed for specific IgG1 response. *In vitro* human studies prove that stimulation by MSP1-_19_ preferentially elicits IL-10 production [33]. Moreover, IL-10 production specific to schistosome antigens was very relevant in children infected by schistosomes [34]. This cytokine is known to favour the production of IgG1 and IgG3 isotypes, especially during malaria infection [33], [35]. In addition, specific IgG3 response has been closely associated with the production of IFN-γ during human infectious diseases, such as *Borrelia burgdorferi* [36] or *S. haematobium* chronic infections [F. Remoue, unpublished data]. In the acute uncomplicated malaria, it has been demonstrated that peripheral blood CD4+ T cells producing both IFN-γ and IL-10 were significantly increased during drug-induced clearance of parasitaemia [37]. Moreover, it has been shown that T cells from vaccinated healthy volunteers and adults naturally exposed to malaria with MSP1-_19_ could produce both IFN-γ and IL-10 [38].

In our study, no statistical correlation of specific IFN-γ or IL-10 productions with IgG1 and/or IgG3 production has been observed. Nevertheless, we have shown an association between the production of these cytokines and the regulation of these specific isotypes according to the group of infection. With regard to previous studies [33]–[37] and the present results, we can suggest that IFN-γ, acting in synergy with IL-10, could be associated to events leading to the predominant IgG3 production as previously observed during others parasitic infections [F. Remoue, unpublished data]. In addition, it is likely that the production of anti-*P. falciparum* IgG1 and IgG3 Abs could be differently regulated by IL-10 and IL-10/IFN-γ, respectively. This hypothesis is now under investigation by analysing the *in vitro* isotype production after cytokine stimulation of T cells from infected individuals.

The influence of environment, genetic background or nutritional status cannot be ruled out to explain the variation of specific immunity observed in our study. Nevertheless, the concomitant infections such as schistosomiasis in children may influence the acquired anti-malaria Ab response and associated cytokine production. The presence of chronic schistosomiasis infection could specifically increase IL-10 and IFN-γ production in coinfected children, a cytokine production which would positively influence the production of cytophilic anti-malaria IgG1 and IgG3 isotypes, known to be protective during malaria infection. Altogether, this study represents an approach of the regulation of anti-malaria blood stage immunity by coinfection and indicates that the helminthic infection status has to be taken into account in the malaria epidemiological and vaccine studies.
